# Frizzled 7 drives amplification of cancer stem-cell subpopulations and the aggressiveness and poor differentiation of human hepatocellular carcinoma

**DOI:** 10.1371/journal.pone.0332768

**Published:** 2025-10-07

**Authors:** Anaïs Lopez, Alexia Paturel, Nadim Fares, Floriane Pez, Guanxiong Wang, Patricia Gifu, Lydie Lefrançois, Jihed Chouaref, Pierre Saintigny, Janick Selves, Jean-Marie Peron, Michel Rivoire, Philippe Merle, Claude Caron de Fromentel

**Affiliations:** 1 INSERM U1052, CNRS-5286, Univ Lyon, Université Claude Bernard Lyon-1, Centre Léon Bérard, Centre de Recherche en Cancérologie de Lyon, Lyon, France; 2 UCLy (Lyon Catholic University), ESTBB, Lyon, France; 3 UCLy (Lyon Catholic University), UR CONFLUNCE: Sciences et Humanités (EA1598), Lyon, France; 4 Département de Médecine Translationnelle, Centre Léon Bérard, Lyon, France; 5 Département d’Anatomie et Cytologie Pathologique, Institut Universitaire du Cancer, Toulouse Oncopôle, Toulouse, France; 6 Service d’Hépato-Gastroentérologie, Hôpital Purpan, Toulouse, France; 7 Département de Chirurgie et Institut de Chirurgie Expérimentale, Centre Léon Bérard, Lyon, France; 8 Hospices Civils de Lyon, Service d'Hépatologie et de Gastroentérologie, Groupement Hospitalier Lyon Nord, Lyon, France; CSIR-Indian Institute of Chemical Biology, INDIA

## Abstract

FZD7 is one of the key players in the subset of WNT-TGFβ-activated hepatocellular carcinomas (HCC), but the consequences of its abnormal expression on hepatocarcinogenesis remain to be better understood. Herein, we aimed to investigate the role of the FZD7-mediated signaling in immature phenotype and aggressiveness of HCC. Firstly, 499 human HCCs were used for clinical and molecular comparisons regarding the expression of *FZD7* and stemness-associated markers. We showed that *FZD7* overexpression was associated with poor differentiation and, in combination with *CD133*, predicted a poor outcome of patients with aggressive recurrence. Next, the impact of WNT3/FZD7 signaling on the differentiation of hepatic cells was assessed in HCC cell lines, as well in the non-transformed progenitor HepaRG cell line and in primary human hepatocytes, transduced with *WNT3* and *FZD7*-expressing lentiviruses. We demonstrated that the ectopic expression of *WNT3* and *FZD7* inhibited the differentiation behavior of HepaRG cells and human primary hepatocytes, amplified the pool of EpCAM^(+)^, CD90^(+)^ and CD133^(+)^ subsets of HCC cell lines, and increased their cancer stem cell features. Moreover, we found that WNT3/FZD7-mediated stemness properties of cancer cells were independent of the stemness-associated marker NANOG. In conclusion, we identified the FZD7^(+)^/CD133^(+)^ signature as a potential prognosis marker and molecular therapeutic target, and we strengthened the hypothesis for the involvement of FZD7 in the enrichment of a cancer stem cell pool in HCC.

## Introduction

Hepatocellular carcinoma (HCC) is one of the main causes of cancer death worldwide [1,2]. WNT/FZD signaling plays an important role in the origin and maintenance of stem cells (SCs) and cancer stem cells (CSCs). These functions are shared with other pathways such as Hedgehog, Notch, and TGF-β/BMP in various tissues and cancer types [3–7]. The WNT/FZD pathway can signal through the canonical β-catenin or the non-canonical PKC and JNK pathways, controlling cell homeostasis and behavior [8]. Regarding hepatocarcinogenesis, deregulation of the WNT/β-CATENIN pathway has been described as a potential key event due to activating *CTNNB1, AXIN1, APC* and *ZNRF3* mutations, or deregulated expression of WNT/FZD complex-receptor elements [9,10]. Furthermore, some classifications clearly identified distinct clusters of HCCs associating both wild-type β- catenin activity and WNT/FZD complex-receptor activation [11–13]. Indeed, Frizzled 7 (FZD7), one of the 10 Frizzled receptors, was found to be overexpressed in some HCCs, activating wild-type β-catenin and functionally increasing survival, proliferation, motility and epithelial-mesenchymal transition [10,14–19]. Importantly, the auto-paracrine WNT3 soluble extracellular factor was clearly identified as a natural canonical activating ligand of FZD7 [17]. These data opened a wide range of perspectives and hope for the targeting of the WNT pathway for the treatment of HCC [9,20].

Herein, we ascertained in human HCC samples whether *FZD7* overexpression correlates with a poor differentiation phenotype and could predict a poor outcome for patients due to tumor aggressiveness. Furthermore, we investigated the implication of canonical WNT3/FZD7-mediated signaling in HCC cell behavior and stemness. We studied its influence on the differentiation program of hepatocytes and liver progenitors, as well as on the amplification of immature HCC sub-populations with stemness and aggressiveness markers. We also assessed the implication of the WNT3/FZD7-mediated signaling for CSC properties. Finally, we investigated the existence of potential cross-talk in HCC CSCs between the WNT3/FZD7 pathway and EpCAM, a SC-associated marker [21–23]. To address these aims, the study was designed in three main steps: first, to analyze the expression pattern of FZD7 and stemness/tumor aggressiveness-associated genes by RT-qPCR in a cohort of HCC samples compared to normal livers; second, to assess the impact of ectopic WNT3/FZD7 signaling on hepatocyte differentiation in PHHs and HepaRG cells; and third, its impact on CSC features through functional assays and stemness marker expression.

## Materials and methods

### Human liver tissues

A collection of 170 frozen human HCC samples, derived from surgically HCC resections was obtained from the PaThLiv French Liver Biobank (Lyon, France, agreement # DC2025–7145), among them 150 with both the tumor (T) and the matched non-tumor liver parenchyma (NT) (at least 2 cm distant of the tumor burden). One hundred and forty samples were evaluable for survival rate analysis (were removed those with liver transplantation post-resection, or early death within 3 months after surgery unrelated to HCC recurrence). Eleven surgically resected non-tumor liver (NL) tissues surrounding colorectal adenocarcinoma metastasis were provided by the Biological Sample Management Plateform (PGEB) of the Centre Léon Bérard, Lyon, France, agreement # BB-0033–00050) and used as a reference. All clinical data linked to the samples were pseudonymized. Histological analysis was performed: i) on T to confirm the diagnosis and characteristics of HCCs; ii) on NT and NL to ensure the absence of microscopic tumor invasion by respectively HCC (for NT) and colon adenocarcinoma metastasis (for NL).

In addition to this first cohort, data of 329 HCCs were obtained from The Cancer Genome Atlas (TCGA), 275 of them being evaluable for survival rates.

### Cell lines and primary human hepatocytes

The Focus (CVCL_7955), Huh7 (CVCL_0336) and HepaRG (CVCL_9720) human cell lines were cultured as previously described [24–26]. The absence of mycoplasma contamination was regularly verified by PCR. Primary Human Hepatocytes (PHHs) were isolated from the resected NLs (see above) as described in [27].

### Plasmids and lentiviral transduction

Human *FZD7* cDNA was cloned into the plenti6/V5-Tag directional TOPO lentivirus containing the blasticidin selection gene (Invitrogen, USA), with an empty vector serving as negative control (EV) [16]. The *WNT3*-HA sequence was extracted from the plenti6/WNT3-HA lentiviral vector [16] and cloned into the pBB/HA lentiviral vector containing the hygromycin B selection gene, with an empty vector as negative control. shFZD7 and shSCR sequences were cloned in the pLKO.1 lentiviral vector containing the puromycin selection gene. Constructs were sequenced in both strands. Virions were produced in HEK293T cells (Invitrogen, USA). Cell lines and PHHs were transduced at a multiplicity of infection of one (MOI-1), as previously described [27].

### Quantitative real-time RT-PCR (RT-qPCR)

Total RNA of cultured cells or frozen tissues was extracted with Extract-all (Eurobio, France), whereas total RNA of FACS-sorted cells was extracted with Nucleospin RNA/XS (Macherey Nagel, France). M-MLV reverse transcriptase (Invitrogen, USA) was used for cDNA synthesis after pre-treatment by DNAse-I (Roche, Switzerland). PCR reactions were performed in the Light Cycler 480 (Roche, Switzerland), with a mix of 1X-Quantifast SYBR Green (Qiagen, Netherlands), 500 nM of each primer and cDNA equivalent to 12.5 ng of total RNA. The thermal cycling conditions comprised an initial step of 5 min at 95°C, followed by 40 cycles of 10 sec at 95°C and 30 sec at 60°C. Each PCR run included standard curves and a non-template control to eliminate remaining contaminating DNA. Analysis was performed using the comparative ∆Ct method, and each gene normalized to the 18S RNA as housekeeping gene [18]. Primers were selected using the Primer3 website (https://primer3plus.com/cgi-bin/dev/primer3plus.cgi), validated, and are available on request. Gene overexpression in T and NT tissues was assessed relative to mean + 2 SD of expression in NL.

### Protein extraction and Western blot analysis

For the detection of excreted WNT3, 200 µL of cell supernatant were taken, heat-denatured and the proteins separated by SDS-polyacrylamide gel electrophoresis (SDS-PAGE). For the detection of membranous FZD7, cells were harvested by scrapping, incubated 30 minutes on ice in lysis buffer [10 mM Tris-HCl pH 7.4, 100 mM NaCl, 1 mM EDTA, 1 mM EGTA, 1 mM NaF, 20 mM Na4P2O7, 2 mM Na3VO4, 1% Triton X-100, 10% glycerol, 0.1% SDS, 0.5% desoxycholate, 10 µL/mL complete protease inhibitor cocktail (Roche, Switzerland)] and sonicated three times for 30 seconds. After centrifugation, protein concentration was measured with the BCA Protein assay kit (Pierce, USA) heat-denatured and separated by SDS-PAGE. Western blot was carried out using the following antibodies: anti-β-ACTIN 1/10,000 (A5316, Sigma, Germany), anti-V5 1/5,000 (R960-25, Invitrogen, USA), anti-HA 1/5,000 (H3663, Sigma, Germany). After adding the rabbit anti-mouse IgG horseradish peroxidase conjugated secondary antibody (A9044, Sigma, Germany), blots were visualized with the enhanced chemiluminescence detection system (Amersham, United Kingdom).

### Immunofluorescence staining

For immunofluorescence (IF), cells were fixed with 4% Paraformaldehyde (PFA), and thereafter permeabilized by pre-chilled methanol for 5 min at room temperature. Slides were incubated for 1 h at room temperature in blocking buffer (PBS 1x, 2 mg/mL BSA, 1% gelatin, 5% goat serum and 0.1% triton X-100), and thereafter incubated with either mouse anti-V5 antibody 1/200 (R690-25, Invitrogen, USA), or rabbit anti-CD133 antibody 1/200 (ab-19898, Abcam, United Kingdom) overnight at 4°C. Alexa488-conjugated goat anti-mouse (150117, Abcam, United Kingdom) or anti-rabbit (150077, Abcam, United Kingdom) secondary antibodies were incubated for 1 h at room temperature.

### Flow cytometry and fluorescence-activated cell sorting (FACS)

Cells were collected and stained as previously described [27]. Briefly, 0.5 x 10^6^ cells in 100 µL of staining buffer (1X PBS, 2% BSA) were incubated on ice for 10 min for non-specific antigen saturation. Staining was performed with an APC-conjugated anti-EpCAM antibody 1/20 (Clone EBA-1 347200, BD Biosciences, USA) or a PerCP Cy5-conjugated anti-CD90 antibody 1/20 (eBio 45–0909, clone 5E10, eBiosciences, USA), according to manufacturer’s instructions. Staining analysis was performed with the FACScan flow cytometer (Becton Dickinson). For fluorescence-activated cell sorting (FACS), after staining with the APC-conjugated anti-EPCAM monoclonal antibody, cells were washed, resuspended in PBS-1X, 2% Fetal Calf Serum (FCS) and sorted by using the BD FACS Aria II cell sorting system (BD Biosciences, USA).

### Clonogenicity and hepatosphere formation assay

For colony formation unit (CFU) assay, 200 EpCAM^(-)^- or EpCAM^(+)^-sorted cells were seeded in 6-well plates, and after 14 days of subsequent culture, were either fixed (20% methanol)/stained (0.5% crystal violet), or fixed (4% PFA)/labeled for IF. Regarding the hepatosphere formation assay, cells were enzymatically harvested and seeded at 50,000 cells/mL in ultra-low attachment 24 well plates (Corning, USA) with DMEM containing 1XB27 supplement (Life Technologies, USA), 20 ng/mL EGF (R&D Systems, USA), 20 ng/mL bFGF (StemCell Technologies, Canada), 4 µg/mL heparin, and 1% penicillin/streptomycin (Invitrogen, USA). Cells were cultured for 3 days at 37°C with 5% CO_2_. Spheres were measured and counted by phase-contrast microscopy.

### Bioinformatics analysis

The liver tissue samples used in this study are the same as those previously processed and described in [28]. The data, normalized gene-read counts generated from RNA-sequencing (RNASeq version 2 – level 3), were downloaded using the TCGA2STAT R-package [29], and were log2 transformed. Prior to the downloading of data, normalization was performed using MapSlice for the alignment and RSEM to perform the quantification [30,31]. Clinical data were retrieved from the cBioPortal database [32,33].

### Statistical analysis

MedCalc Version 12.7.1.0 software was used for statistical analysis. Continuous variables and proportions were compared using the Mann-Whitney (Wilcoxon for paired samples), *t-*Student (paired samples *t*-test when appropriate), and Chi-squared or Fischer’s exact tests. Survival was assessed by the Cox proportional-hazards regression in univariate and multivariate analysis, hazard ratios (HR) with 95% confidence intervals (CI), and *p* values were calculated using the log-rank (Mantel–Cox) test. Tests were considered significant when *p* values were < 0.05. Unsupervised hierarchical clustering with dendrogram was performed with NCSS 10 software.

## Results

### Clinicopathological correlations and prognosis value of FZD7-related signatures in HCC

The expression pattern of *FZD7* and genes associated with stemness and tumor aggressiveness (*EPCAM*, *NANOG*, and *CD133*) were analyzed by RT-qPCR in an initial cohort of 170 surgically resected HCCs and compared to a panel of normal livers (NLs). When found overexpressed, the genes were annotated *FZD7*^(+)^, *EPCAM*^(+)^, *NANOG*^(+)^ and *CD133*^(+)^. *EPCAM*^(+)^ populations strongly correlated with poor differentiation of HCCs, AFP level > 200 ng/mL and HCC multinodularity, whereas *NANOG*^(+)^ correlated with the appearance of extra-hepatic recurrence (Table 1). For *FZD7*^(+)^, a close link was found with the poor differentiation of tumors (Table 1) and confirmed by integrating 329 additional HCCs of the TCGA cohort (total HCCs, *n = *499) in the analysis (S1 Table).

**Table 1:** Prevalence of *FZD7, EPCAM, NANOG* and *CD133* overexpression in a French cohort of 170 HCCs regarding clinicopathological and biological parameters. Chi-squared test (*p* value). *p* value < 0.05 was considered as significant (in bold). AFP, α-fetoprotein level; HBV, hepatitis B virus; HCV, hepatitis C virus; NASH, non-alcoholic steato-hepatitis;). ^(+)^, overexpression.

	*FZD7* ^(+)^	*EPCAM* ^(+)^	*NANOG* ^(+)^	*CD133* ^(+)^
Total prevalence	39%	33%	30%	21%
Etiology: HBV *vs.* HCV *vs.* alcohol *vs.* NASH vs. others (p)	53% *vs.* 34% *vs.* 42% *vs.* 36% *vs.* 38% *(0.13)*	40% *vs.* 52% *vs.* 32% *vs.* 36% *vs.* 23% *(0.23)*	37% *vs.* 32% *vs.* 25% *vs.* 21% vs. 36% *(0.65)*	27% *vs.* 28% *vs.* 16% *vs.* 14% *vs.* 18% *(0.18)*
Presence *vs.* absence of cirrhosis (p)	58% *vs.* 35% *(0.34)*	35% *vs.* 30% *(0.49)*	29% *vs.* 31% *(0.80)*	16% *vs.* 25% *(0.16)*
Tumor size > 50 mm *vs.* ≤ 50 mm (p)	39% *vs.* 44% *(0.19)*	30% *vs.* 35% *(0.47)*	34% *vs.* 27% *(0.30)*	17% *vs.* 25% *(0.23)*
Presence *vs.* absence of HCC multinodularity (p)	43% *vs.* 39% *(0.71)*	54% *vs.* 28% ***(0.003)***	37% *vs.* 29% *(0.34)*	26% *vs.* 20% *(0.50)*
Poor *vs.* moderate *vs.* good differentiation (p)	71% *vs.* 36% *vs.* 31% ***(0.002)***	50% *vs.* 37% *vs.* 22% ***(0.006)***	38% *vs.* 19% *vs.* 32% *(0.35)*	33% *vs.* 16% *vs.* 19% *(0.24)*
AFP > 200 *vs.* ≤ 200 ng/mL (p)	45% *vs.* 40% *(0.57)*	58% *vs.* 30% ***(0.002)***	42% *vs.* 28% *(0.11)*	24% *vs.* 22% *(0.82)*
Presence *vs.* absence of microvascular invasion (p)	43% *vs.* 38% *(0.53)*	40% *vs.* 29% *(0.15)*	36% *vs.* 24% *(0.10)*	19% *vs.* 23% *(0.51)*
Presence *vs.* absence of satellite nodules (p)	44% *vs.* 39% *(0.57)*	44% *vs.* 30% *(0.08)*	33% *vs.* 28% *(0.57)*	17% *vs.* 23% *(0.37)*
Recurrence: extra-hepatic *vs.* hepatic multifocal *vs.* hepatic uninodular (p)	49% *vs.* 38% *vs.* 44% *(0.63)*	41% *vs.* 36% *vs.* 28% *(0.60)*	46% *vs.* 26% *vs.* 16% ***(0.009)***	27% *vs.* 14% *vs.* 32% *(0.19)*

Among the 170 HCC patients of the initial cohort, only 140 were evaluable for survival analysis with the baseline clinicopathological characteristics described in S2 Table, showing a balanced percentage between viral and non-viral etiologies, and a majority of patients with little impaired liver functions (Child-Pugh A) and BCLC-A stage tumor (S2 Table). As expected, in univariate analysis, recurrence-free survival (RFS) was worsened by the presence of satellite nodules, microvascular invasion, multinodularity, tumor size > 50 mm, and underlying cirrhosis in univariate analysis (Table 2, upper panel). In multivariate analysis, only microvascular invasion, tumor size > 50 mm, and cirrhosis remained significant (Table 2, lower panel).

**Table 2:** Correlation between survival rates and stemness/clinicopathological features in 140 HCCs of the French cohort. Cox proportional-hazards regression in univariate (upper panel) and multivariate analysis (lower panel); hazard ratios (HR) with 95% confidence intervals (CI). *p* value < 0.05 was considered as significant (in bold).). ^(+)^, overexpression.

Univariate analysis of survivals regarding variables *vs.* rest	Overall survival (OS)		Recurrence-free survival (RFS)		Post-recurrence survival	
	HR (95% CI)	*p*	HR (95% CI)	*p*	HR (95% CI)	*p*
Tumor size > 50 mm	0.80 (0.42–1.53)	*0.50*	1.55 (1.01 - 2.41)	***0.04***	0.58 (0.30 - 1.11)	*0.09*
HCC multinodularity	1.89 (0.96 - 3.74)	*0.06*	1.65 (1.01 - 2.69)	***0.04***	1.40 (0.70 - 2.77)	*0.34*
Poor tumor differentiation	0.75 (0.27 - 2.12)	*0.59*	1.22 (0.66 - 2.25)	*0.53*	0.71 (0.25–2.02)	*0.52*
AFP > 200 ng/mL	1.19 (0.60 - 3.31)	*0.68*	1.09 (0.60–1.97)	*0.78*	1.14 (0.50 - 2.61)	*0.75*
Microvascular invasion	1.64 (0.87–3.09)	*0.12*	1.82 (1.16 - 2.85)	***0.008***	1.21 (0.64–2.28)	*0.55*
Satellite nodules	2.72 (1.37–5.38)	***0.004***	1.66 (1.01–2.72)	***0.04***	2.22 (1.11–4.41)	***0.02***
Extrahepatic recurrence	1.72 (0.88–3.41)	*0.11*	1.41 (0.86–2.32)	*0.17*	1.61 (0.81 - 3.20)	*0.17*
Cirrhosis	3.71 (1.76–7.86)	***0.0006***	1.66 (1.05–2.63)	***0.03***	2.82 (1.33–5.95)	***0.006***
*FZD7* ^*(+)*^	1.01 (0.53–1.90)	*0.99*	1.14 (0.73–1.76)	*0.57*	1.04 (0.55 - 1.98)	*0.90*
*EPCAM* ^*(+)*^	1.96 (1.04–3.36)	***0.03***	1.15 (0.72–1.83)	*0.56*	2.17 (1.15–4.10)	***0.01***
*NANOG* ^*(+)*^	1.50 (0.79 - 2.84)	*0.22*	1.07 (0.67–1.72)	*0.76*	1.50 (0.80 - 2.85)	*0.22*
*CD133* ^*(+)*^	1.75 (0.89 - 3.45)	*0.10*	1.25 (0.74–2.12)	*0.39*	1.88 (0.95 - 3.70)	*0.07*
*FZD7* ^*(+)*^ */ EPCAM* ^*(+)*^	1.43 (0.63 - 3.23)	*0.39*	1.11 (0.61–2.00)	*0.73*	1.67 (0.73–3.82)	*0.23*
*FZD7* ^*(+)*^ */ NANOG* ^*(+)*^	1.95 (0.93 - 4.11)	*0.08*	1.15 (0.63 - 2.09)	*0.64*	2.22 (1.04–4.71)	***0.03***
*FZD7* ^*(+)*^ */ CD133* ^*(+)*^	2.38 (1.13 - 5.02)	***0.02***	1.05 (0.55–1.98)	*0.89*	3.57 (1.66–7.68)	***0.001***
*EPCAM*^*(+)*^*/NANOG* ^*(+)*^	2.19 (1.01 - 4.77)	***0.04***	0.94 (0.47–1.90)	*0.88*	3.03 (1.38–6.65)	***0.005***
*EPCAM* ^*(+)*^ */ CD133* ^*(+)*^	1.48 (0.66–3.36)	*0.34*	1.10 (0.58 - 2.08)	*0.77*	1.82 (0.80 - 4.14)	*0.15*
*NANOG* ^*(+)*^ */ CD133* ^*(+)*^	2.83 (1.19–6.77)	***0.01***	1.41 (0.65–3.07)	*0.38*	3.39 (1.40 - 8.20)	***0.006***
**Multivariate analysis of survivals regarding variables *vs.* rest**	**Overall survival**		**Recurrence-free survival**		**Post-recurrence survival**	
	**HR (95% CI)**	***p***	**HR (95% CI)**	***p***	**HR (95% CI)**	***p***
Tumor size > 50 mm			1.77 (1.06–2.95)	***0.02***		
HCC multinodularity			1.50 (0.90–2.52)	*0.12*		
Microvascular invasion			1.69 (1.04–2.76)	***0.03***		
Satellite nodules	2.43 (1.21–4.88)	***0.01***	0.96 (055–1.58)	*0.88*	2.12 (1.03–4.34)	***0.04***
Cirrhosis	3.41 (1.54–7.55)	***0.002***	2.44 (1.43–4.17)	***0.001***	2.38 (1.07–5.31)	***0.03***
*FZD7* ^*(+)*^ */ CD133* ^*(+)*^	2.38 (1.03 - 5.05)	***0.04***			2.22 (1.02–5.09)	***0.04***
*EPCAM* ^(+)^	1.69 (0.85 - 3.35)	*0.13*			1.42 (0.69–2.93)	*0.34*

Regarding overall survival (OS) in univariate analysis, satellite nodules and cirrhosis parameters correlated with poor outcome, as well as some stemness signatures, such as *EPCAM*^(+)^, *FZD7*^(+)^/*CD133*^(+)^, *EPCAM*^(+)^/*NANOG*^(+)^, and *NANOG*^(+)^/*CD133*^(+)^. The recurrent presence of *NANOG* in signatures suggested its potential key role in stemness. Interestingly, poor OS did not correlate to shorter RFS but rather to shorter survival after tumor recurrence, suggesting these recurrences as being enriched in stem-like cells and harboring an aggressive phenotype (Table 2, upper panel). In the multivariate analysis, some molecular signatures (*NANOG*^*(+)*^*/CD133*^(+)^ and *EPCAM*^*(+)*^*/NANOG*^*(+)*^) were excluded from Table 2 (lower panel) because, as shown in the heat map (S1 Fig), the majority of the *NANOG*^*(+)*^*/CD133*^*(+)*^ cluster is incorporated into the larger *FZD7*^*(+)*^*/CD133*^*(+)*^ cluster, and the *EPCAM*^*(+)*^*/NANOG*^*(+)*^ cluster into the larger *EPCAM*^*(+)*^ cluster. Furthermore, in multivariate analysis, only satellite nodules, cirrhosis and the *FZD7*^(+)^/*CD133*^(+)^ signature impacted on poor OS through shorter post-recurrence survival (Table 2, lower panel).

All of the analyzed stemness signatures impacting on OS correlated between each other and could not be identified as independent variables in multivariate analysis (Rho Spearman's coefficient of rank correlation, *p* value), for example, *FZD7*^(+)^/*NANOG*^*(+)*^ **vs.* FZD7*^(+)^/*CD133*^(+)^ (0.409, *p < *0.0001), *FZD7*^(+)^/*NANOG*^*(+)*^ **vs.* EPCAM*^(+)^/*NANOG*^(+)^ (0.513, *p < *0.0001), *FZD7*^(+)^/*NANOG*^*(+)*^ **vs.* NANOG*^(+)^/*CD133*^(+)^ (0.661, *p = *0.0001), *FZD7*^(+)^/*CD133*^(+)^ **vs.* EPCAM*^(+)^/*NANOG*^(+)^ (0.399, *p < *0.0001), or *FZD7*^(+)^/*CD133*^(+)^ **vs.* NANOG*^(+)^/*CD133*^(+)^ (0.682, *p < *0.0001) (S3 Table). In addition to *FZD7*^(+)^/*CD133*^(+)^ that appeared independent in multivariate analysis, *NANOG*^*(+)*^ seemed to be a key player, present in all stemness signatures with predictive outcome.

The addition of 275 samples from the TCGA cohort evaluable for survival analysis allowed confirmation in a total of 415 HCCs (140 + 275) that the *FZD7*^(+)^/*CD133*^(+)^ signature predicted poorer OS associated with rapid death after recurrence, while the *NANOG*^(+)^/*CD133*^(+)^ signature predicted a poorer OS, independently of both rapid death after recurrence and RFS (S4 Table). Of note, the *FZD7*^(+)^/*NANOG*^(+)^ signature was not significant to predict OS. This observation suggests that FZD7 and NANOG affect patient outcome through independent pathways.

### WNT3/FZD7-mediated signaling impairs hepatocyte differentiation

**Fig 1:**
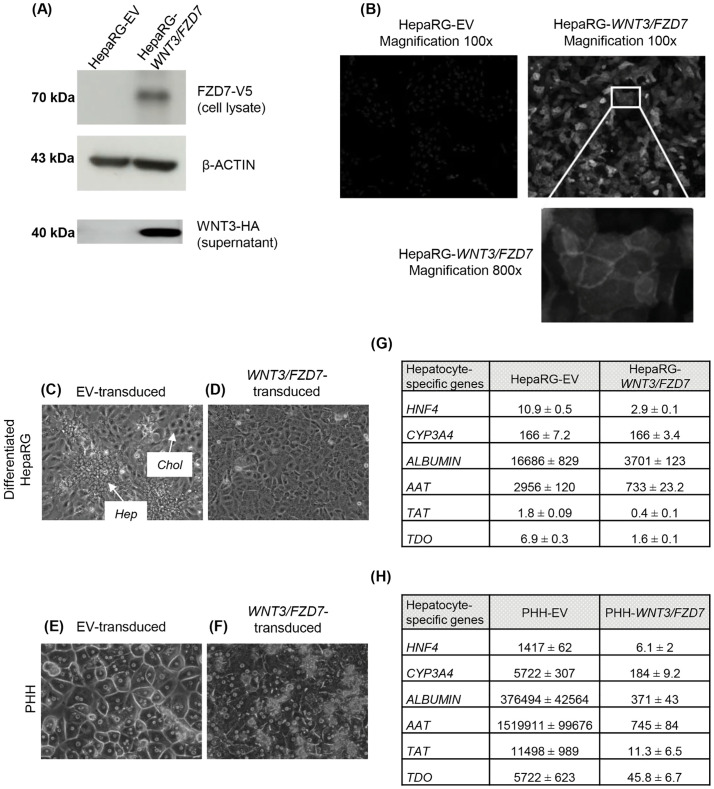
The expression of *FZD7* and *WNT3* transgenes in HepaRG cells and PHHs affects hepatocyte differentiation. Western blot of HepaRG cells transduced with the combination of *FDZ7* and *WNT3* lentiviruses or with the corresponding empty vectors (EV). *FZD7-V5* tagged expression was assessed in cell lysate and *WNT3-HA* tagged in supernatant, respectively (A). Uncropped blots are shown on S1 Raw Images. Immunofluorescence showing FZD7 localization at the cell membrane of HepaRG cells (magnifications 100x and 800x) (B). HepaRG-EV-transduced cells reached terminal differentiation into hepatocyte and cholangiocyte-like cells (C) at confluence, in contrast to HepaRG-WNT3/FZD7-transduced cells (D). Freshly isolated PHHs were platted at high density and subsequently transduced with EV (E) or *WNT3/FZD7* lentiviruses (F). Phase-contrast microscopy examination of cells is shown, pictures taken in randomly selected fields representative of three independent experiments (magnification, 100x for C and D, 200x for E and F). Expression of hepatocyte differentiation markers assessed by RT-qPCR after three weeks of culture and antibiotic selection in three independent experiments in HepaRG (G) and PHH (H).

Since *FZD7*^*(+)*^ correlated with a poor differentiation status in HCC cohorts, we assessed whether FZD7, canonically activated by WNT3 in an experimental setting, could reverse the terminal differentiation of PHHs (constitutively differentiated quiescent hepatocytes), and/or inhibit the differentiation process occurring in HepaRG progenitors when reaching confluence (proliferating liver progenitors when plated at low density, and thereafter differentiating into quiescent hepatocytes and cholangiocytes at confluence) [34]. Transgene expression was confirmed by Western blot in cell lysate for FZD7 and in supernatant for excreted WNT3 (Fig 1A) and FZD7 localization at the cell membrane by IF (Fig 1B). Then, HepaRG, cells were transduced when proliferating at low density, and then cultured under conditions for terminal differentiation. Striking morphological differences were observed at the end of the differentiation process after four weeks at confluence (Fig 1C and 1D). Like the parental HepaRG cells previously described [34], HepaRG-EV cells gave evidence for a bidirectional differentiation into hepatocyte (Hep) and cholangiocyte (Chol) clusters (Fig 1C). In contrast, HepaRG-WNT3/FZD7 cells did not establish any structured monolayer, and none of the morphologic criteria of hepatocytes or cholangiocytes was observed (Fig 1D). Regarding PHH, phase-contrast microscopy showed cellular stress after lentiviral delivery of WNT3/FZD7 *vs.* EV (Fig 1E and 1F). However, PHH-EV, PHH-WNT3/FZD7 did not enter the cell cycle and remained in a non-proliferative state.

Most of the hepatocyte differentiation markers observed in differentiated HepaRG cells and in PHH (*HNF4, CYP3A4, ALBUMIN, AAT, TAT, TDO*) drastically decreased in HepaRG-WNT3/FZD7 (Fig 1G) and in PHH-WNT3/FZD7 (Fig 1H) *vs.* their respective EV. These results suggested that WNT3/FZD7 could impair the differentiation status of terminally differentiated PHH and the differentiation dynamics of HepaRG cells.

### WNT3/FZD7-mediated signaling enhances immature phenotype in HCC Cell lines

The Focus and Huh7 HCC cell lines do not have any identified mutation amongst the components of the canonical WNT pathway (wild-type status of *CTNNB1*, *AXIN1* and *APC* genes) [35]. They are relevant paradigms, expressing moderate levels of endogenous *FZD7* that drives a steady state level of WNT/β-catenin activity, that is easily up- or down-regulated in experimental settings [17,18]. Activation of canonical WNT3/FZD7-mediated signaling in transduced cells (intracellular accumulation of β-catenin and overexpression of the β-catenin-target genes (*CYCLIN-D1* and *AXIN2*)) was previously described [36].

**Fig 2:**
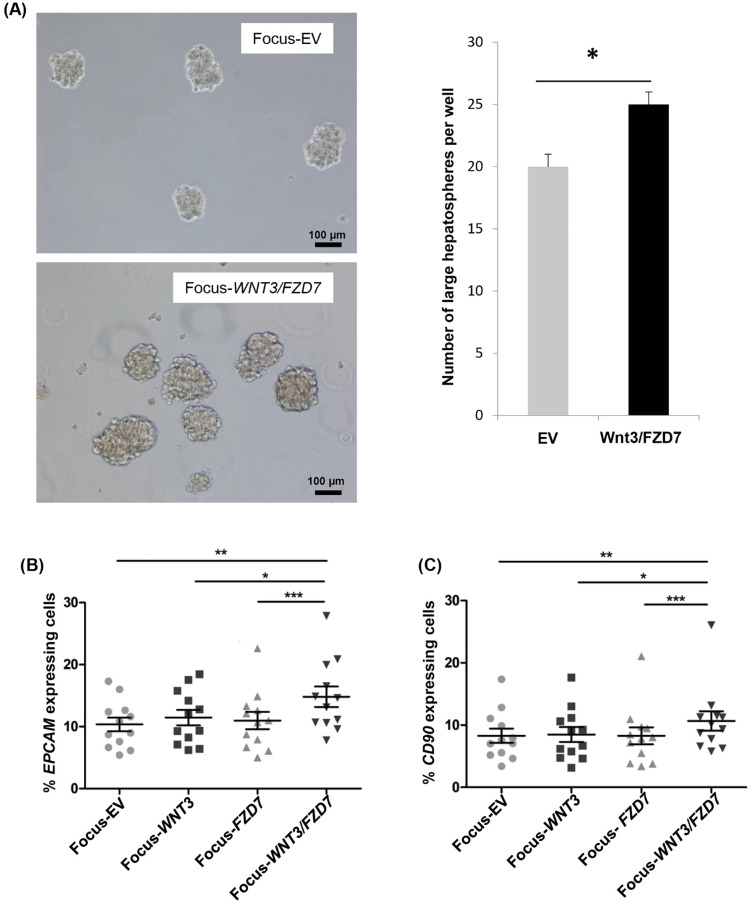
The overexpression of *WNT3/FZD7* increases hepatosphere formation. Focus cells transduced with empty (EV) or *WNT3/FZD7* lentiviruses were cultured in non-adherent conditions for three days. Hepatospheres larger than 100 µm were counted in each well (2 independent experiments in duplicates). Bars represent 100 µm. Paired *t*-test, (*) *p* < 0.05 (A). Increased expression of stemness-associated markers in *WNT3/FZD7*-overexpressing Focus cells. The percentage of Focus-EV, Focus-WNT3, Focus-FZD7 and Focus-WNT3/FZD7 cells expressing EpCAM (B) or CD90 (C) markers was determined by flow cytometry. Data are representative of 12 independent experiments. Bars represent means. Mann-Whitney test: (*) *p <* 0.05, (**) *p <* 0.01, (***) *p <* 0.001.

We evaluated the impact of ectopic WNT3/FZD7 on CSC properties by functional assays and expression of stemness-associated markers. The hepatosphere formation assay showed that Focus-WNT3/FZD7 cells were able to form a significantly higher number of large hepatospheres (>100 µm) than Focus-EV cells, suggesting an enrichment of CSC sub-populations in the Focus-WNT3/FZD7 cells (Fig 2A). CSCs, a small subset of cancer cells in both HCC tumor bulks and cell lines, can be detected by different classical stemness-associated markers such as EpCAM, CD90, and CD133 [37–39]. While the expression of either *WNT3* or *FZD7* alone did not result in significant changes, the *WNT3/FZD7* combination amplified by 42% and 29% the pool of EpCAM^(+)^ and CD90^(+)^ cells, respectively, as compared to Focus-EV cells (Fig 2B and 2C and S5 Table, upper panel), but did not impact the pool of CD133^(+)^ cells (S5 Table, upper panel). Similar data were found with the Huh7 cell line regarding EpCAM and CD90 markers (S5 Table, upper panel). To test the hypothesis for the involvement of FZD7 in this enrichment of EpCAM^(+)^ cells, we invalidated FZD7 by lentivirus-delivery of shRNA. Indeed, the resulting Focus-EV/shFZD7 and Focus-WNT3/FZD7/shFZD7 cells displayed a significant decrease in EpCAM^(+)^ sub-populations (S5 Table, lower panel).

**Fig 3:**
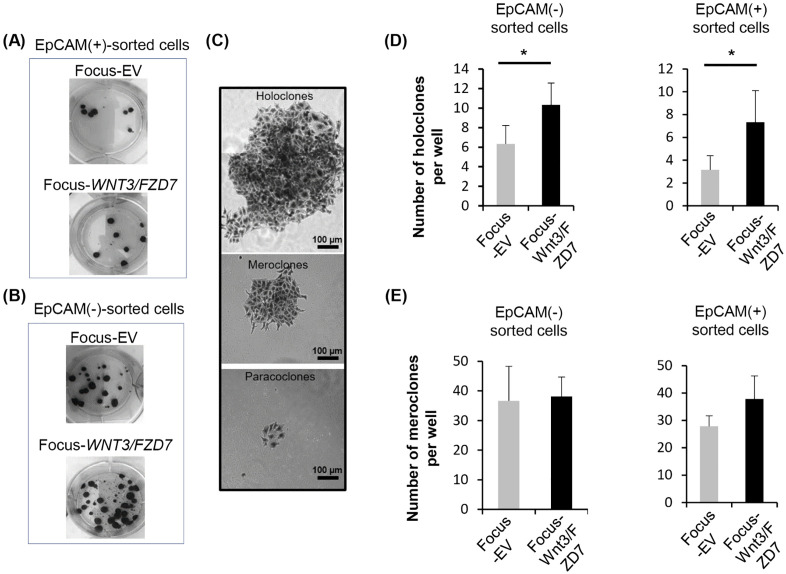
Effect of WNT3/FZD7 overexpression on the immature phenotype of Focus cells. Representative images of CFU formation of FACS-sorted EpCAM^(+)^ (A) and EpCAM^(-)^ (B) sub-populations of Focus-EV and Focus-WNT3/FZD7 cells. The arising colonies exhibited three morphological types termed holo-, mero-, and paraclones. Of note, to obtain a full-length image of the holoclone, three microscopic pictures were merged (C). Holoclones (D) and meroclones (E) were counted for EpCAM^(+)^ and EpCAM^(-)^ cell subpopulations (2 independent experiments in duplicates). Mann-Whitney test, (*) *P <* 0.05.

To highlight potential differences of CSC properties between some subsets of Focus cells, EpCAM^(+)^ and EpCAM^(-)^ sub-populations were separated by FACS and then tested for their capability to form colonies in CFU assay (Fig 3A and 3B). Based on differences in macroscopic and microscopic morphology, colonies were defined as holoclones, meroclones and paraclones as previously described [40] (Fig 3C). Holoclones, which contain tumor initiating cells, are large clusters of homogeneous, small and tightly packed cells with regular and smooth colony borderlines. Paraclones consist of few, dispersed and larger cells, whereas meroclones exhibit intermediate morphologies. Para- and meroclones are composed of late and early transit amplifying cells, respectively, and are devoid of tumor initiating cells. Ectopic expression of *WNT3/FZD7* in Focus cells significantly increased the number of holoclones *per* well in both EpCAM^(+)^ and EpCAM^(-)^ cells (Fig 3D), but not those of meroclones (Fig 3E). Paraclones were not counted since they lack stemness or aggressiveness significance. Interestingly, CD133 intracellular levels, assessed by IF in holoclones arising from EpCAM^(+)^ and EpCAM^(-)^ cells, were similar in the EpCAM^(-)^ sub-populations from EV or WNT3/FZD7 cells (S2A Fig), but strongly increased only in the EpCAM^(+)^ sub-population under WNT3/FZD7 pressure (S2B Fig). These data confirmed that WNT3/FZD7-mediated signaling amplified a pool of cells with CSC properties.

## Discussion

Hepatocarcinogenesis is a heterogeneous and only partially understood process [41,42]. Genetic and transcriptomic classifications have identified clusters of HCCs with activation of wild-type β-catenin associated with overexpression of *FZD7* [11,12,43]. Activation of the canonical WNT pathway non-related to mutations (*CTNNB1*, *AXIN1, APC or ZNRF3*) but rather to transcriptomic changes, such as *FZD7* overexpression, drives embryonic programs [44], but is also found in HCC subtypes resembling hepatic progenitor/stem cells and might be a key player involved in addiction loops for tumor cell survival [45]. The main molecular subclasses of HCC are derived from previously reported transcriptomic based classifications and our *FZD7*-overexpressing tumors very likely belong to the proliferative group, specifically within the G2-G3 subgroups, S1/cluster 3 “TGF-β/WNT”, shown to be associated with a global DNA hypomethylation status, as reviewed by Llovet and collaborators [12].

In the present study, we show that canonical WNT3/FZD7-mediated signaling could inhibit the differentiation program in hepatocytes, amplify the pool of CSCs and drive the aggressiveness of HCC and contribute to poor outcomes of patients. In our experimental strategy, we have used WNT3 as an ectopic ligand for FZD7, since it has been reported to be able to activate the canonical WNT pathway [17,36], keeping in mind that other ligands such as WNT5a can activate FZD7-mediated signaling, mainly non-canonically, as reported in breast cancer [46]. Other membrane complex-receptor components, such as the binding of Cripto-1 to FZD7, are also key drivers of stemness in HCC [47].

Herein, we observed that *FZD7* up-regulation closely correlates to poor differentiation of human HCCs. To demonstrate that FZD7 could directly drive the dedifferentiation program, we used human non-tumorigenic liver cells, such as HepaRG progenitors and PHHs. We demonstrated that the WNT3/FZD7 combination inhibits the commitment of the HepaRG progenitors towards hepatocyte differentiation, but also represses some genes specific for terminal hepatocyte differentiation in PHHs. We then assessed the specific role of WNT3/FZD7 in HCC cell lines. In particular, we looked at several markers previously reported to be overexpressed in liver SCs and CSCs [48], such as *CD90*, *CD133* and *EPCAM* [49,50]. We confirmed that WNT3/FZD7 enhances some stemness features, by amplifying the pool of EpCAM^(+)^ and CD90^(+)^ subpopulations, and the pool of CD133^(+)^ in the EpCAM^(+)^ subset only. We showed in human HCC tissues that *FZD7*^(+)^ closely correlates with *EPCAM*^(+)^ and *CD133*^(+)^, as well as with the poor differentiation status of HCCs. CD133^(+)^ HCC cells were reported to exhibit high proliferative and clonogenic capabilities *in vitro*, and were more prone to *in vivo* tumorigenesis [39,51]. *FZD7* was previously described as overexpressed in HCC cell lines such as Huh7, Focus, and in side populations from HCC cell lines such as PLC/PRF/5 [18,52]. Interestingly, it has also been suggested that FZD7 could mediate CSC self-renewal and EMT in gastric cancer, *via* the canonical WNT signaling pathway [53].

To go further in the implication of WNT/FZD signaling for the stemness features of HCC, we explored the expression of *FZD7* and *CD133*, *EPCAM*, and *NANOG* in human HCC tissues, all of them already associated to CSCs in HCC [9,49,50,54]. When separately evaluated, none of them gave *per se* any prognostic value on survival rates in both univariate and multivariate analyses. The poor prognosis value of *CD133*^(+)^ was only present in association with *FZD7*^(+)^. Indeed, the *FZD7*^(+)^/*CD133*^(+)^ combination signature was strongly associated with poor OS due to rapid death after recurrence of HCC, this remaining true in multivariate analysis. This is in accordance with our experimental setting where canonical FZD7-mediated signaling nonetheless amplified some specific cell subsets with specific stemness markers, but also enhanced the CSC properties of HCC cell lines as assessed by hepatosphere and holoclone formation in functional assays. This was very likely driven by epigenetic reprogramming of HCC cells that governs their malignant properties as previously shown [55].

In summary, some clusters of human HCCs clearly appear to be driven by the canonical WNT pathway in the absence of any mutation in this signaling cascade. Herein, we show that the canonical FZD7-mediated signaling can play a key role in amplifying some subsets of cells with stemness markers among which, some of them harbor CSC properties. We speculate that this process controls a poor differentiation phenotype. Finally, we show that the presence of both FZD7 and CD133 markers in HCCs confers a value of poor prognosis due to more aggressive recurrence. This co-expression profile could be integrated into personalized medicine strategies, enabling more accurate patient stratification and the tailoring of targeted therapies based on individual tumor biology [56,57].

### Novelty and impact

This study highlights the critical role of FZD7 in hepatocellular carcinoma (HCC) aggressiveness by promoting cancer stem cell properties. We demonstrate that *FZD*7 overexpression enhances stemness features independently of *NANOG* and, in association with *CD133* overexpression, predicts poor patient outcome. These findings establish the *FZD7*^*(+)*^*/CD133*^*(+)*^ signature as a potential prognostic marker and therapeutic target, offering new insights into WNT/FZD signaling-associated HCC and opening perspectives for precision medicine strategies.

## Supporting information

S1 Fig Expression patterns of *FZD7*, *CD133*, *NANOG*, and *EPCAM* in human HCC samples. Gene expression was assessed by RT-qPCR in 140 HCCs from the French cohort evaluable for survival rates. For each gene *per* sample, data were centered on a panel of 11 NLs as described in Materials and Methods, and gene considered as upregulated for values > mean + 2 SD (red boxes), or non-upregulated for values ≤ mean + 2 SD (black boxes). Dendrogram of rows is presented on the right, with hierarchical clustering using the Ward’s minimum variance method, Euclidean distance type, range scale type (NCSS 11 software).
(TIF)

S2 Fig CD133 expression in Focus-EV and Focus-WNT3/FZD7 EpCAM^(-)^ and EpCAM^(+)^-sorted cells. Holoclones from EpCAM^(-)^ (A) and EpCAM^(+)^ (B) sub-populations were tested for CD133 expression by IF (magnification 200x and 800x).
(TIF)

S1 Raw Image Uncropped blots corresponding to Fig 1A. (PDF)

S1 Table Prevalence of FZD7 overexpression in the 499 HCCs depending of the differentiation of tumors. 170 HCCs from the French and 329 from the TCGA cohorts were analyzed. Chi-squared test (p value). (+), overexpression.
(DOCX)

S2 Table Clinicopathological characteristics of human HCCs. 140 HCCs were evaluable for survival rates in the French cohort. AFP, α-fetoprotein level; BCLC, Barcelona-Clinic Liver Cancer.
(DOCX)

S3 Table Correlations between stemness signatures influencing overall survival (OS). Data are expressed as Spearman’s rank correlation coefficient (Rho) and p value. All these signatures correlated with each other and could not be identified as independent variables in multivariate analysis.
(DOCX)

S4 Table Correlation between survival rates and the overexpression of FZD7, NANOG and CD133 stemness markers in HCCs. 415 HCCs (140 from the French and 275 from the TCGA cohorts) were analyzed. Cox proportional-hazards regression in univariate analysis; hazard ratios (HR) with 95% confidence intervals (CI). p value < 0.05 was considered as significant (in bold). Stemness signatures correlated between each other by Spearman's coefficient of rank correlation (rho, p value). (+), overexpression.
(DOCX)

S5 Table Expression of stemness-associated markers in WNT3/FZD7-overexpressing Focus and Huh7 cells. Upper panel: The percentage of EpCAM or CD90 positive cells was assessed by FACS in Focus-EV, Focus-WNT3/FZD7 (12 independent experiments), Huh7-EV and Huh7-WNT3/FZD7 (3 independent experiments in duplicate). The percentage of CD133 positive cells was assessed by IF in Focus-EV and Focus-WNT3/FZD7. Ten random fields were analyzed in three independent experiments. Lower panel: The percentage of EpCAM(+) cells was also evaluated in Focus-EV and Focus-WNT3/FZD7, after shRNA-mediated invalidation of FZD7 (5 independent experiments). ND, non-determined. p value < 0.05 was considered as significant (in bold). shSCR, sh scramble.
(DOCX)

## Abbreviations

AFP: α-fetoprotein
BCLC: Barcelona-Clinic Liver Cancer Staging Classification
CFU: Colony formation unit
Chol: Cholangiocytes
CI: Confidence intervals
CSCs: Cancer stem cells
ESCs: Embryonic stem cells, EV, Empty vector
FACS: Fluorescence-activated cell sorting
FCS: Fetal Calf Serum
bFGF: basic fibroblast growth factor
FZD: Frizzled
FZD7; HCC: Hepatocellular carcinoma
Hep: Hepatocytes
HR: Hazard ratios
IF: Immunofluorescence
RT-qPCR: Reverse transcription-quantitative PCR
NL: Normal liver
NT: Non-tumor
OS: Overall survival
PFA: Paraformaldehyde
PHH: Primary human hepatocytes
RFS: Recurrence-free survival
SCs: Stem cells
SD: Standard deviation
SDS-PAGE: SDS-polyacrylamide gel electrophoresis
shRNA: small hairpin RNA
shSCR: sh Scramble
T: Tumor
TCGA: The Cancer Genome Atlas.
