# Supplementary figures and images for: Frizzled 7 drives amplification of cancer stem-cell subpopulations and the aggressiveness and poor differentiation of human hepatocellular carcinoma
Source: PLoS One. 2025 Oct 7;20(10):e0332768. doi: 10.1371/journal.pone.0332768 (PMC12503320; doi:10.1371/journal.pone.0332768)

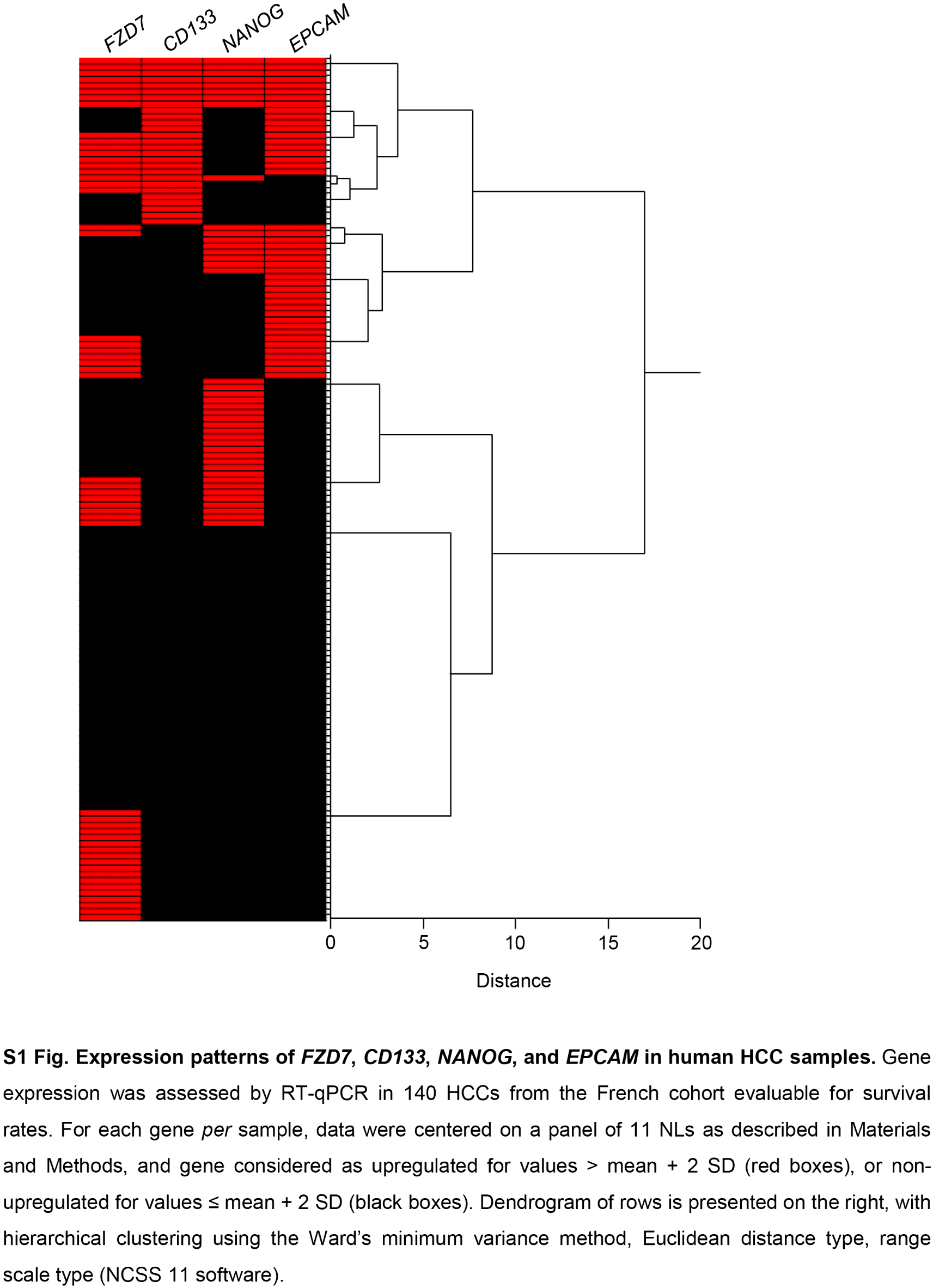

Supplement: S1 Fig — Gene expression was assessed by RT-qPCR in 140 HCCs from the French cohort evaluable for survival rates. For each gene per sample, data were centered on a panel of 11 NLs as described in Materials and Methods, and gene considered as upregulated for values > mean + 2 SD (red boxes), or non-upregulated for values ≤ mean + 2 SD (black boxes). Dendrogram of rows is presented on the right, with hierarchical clustering using the Ward’s minimum variance method, Euclidean distance type, range scale type (NCSS 11 software). (TIF) [file pone.0332768.s001.tif]

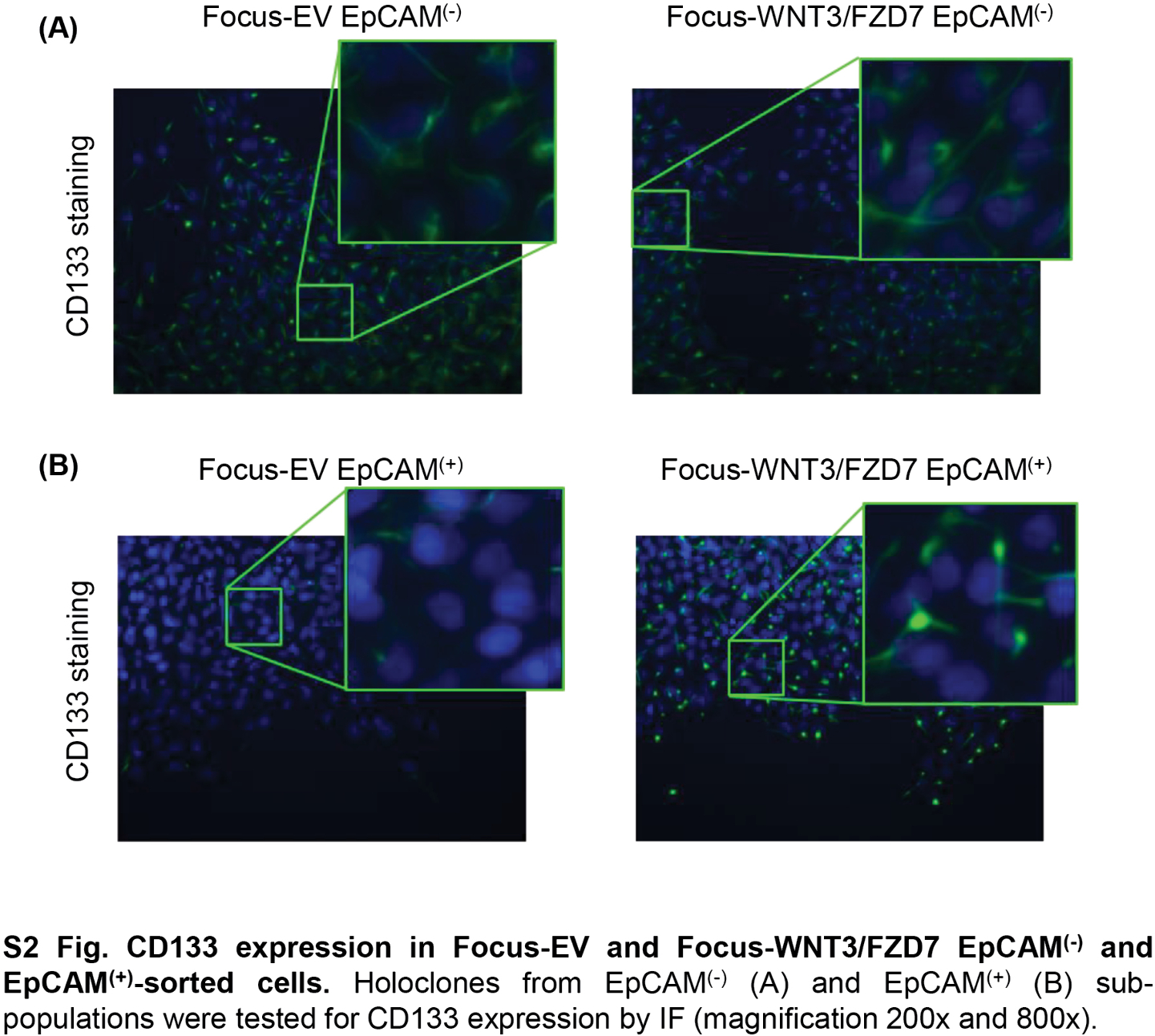

Supplement: S2 Fig — Holoclones from EpCAM(-) (A) and EpCAM(+) (B) sub-populations were tested for CD133 expression by IF (magnification 200x and 800x). (TIF) [file pone.0332768.s002.tif]
