# Supplementary material for: Frizzled 7 drives amplification of cancer stem-cell subpopulations and the aggressiveness and poor differentiation of human hepatocellular carcinoma
Source: PLoS One. 2025 Oct 7;20(10):e0332768. doi: 10.1371/journal.pone.0332768 (PMC12503320; doi:10.1371/journal.pone.0332768)

Autoradiography of blots Figure 1A

Figure 1A upper panel

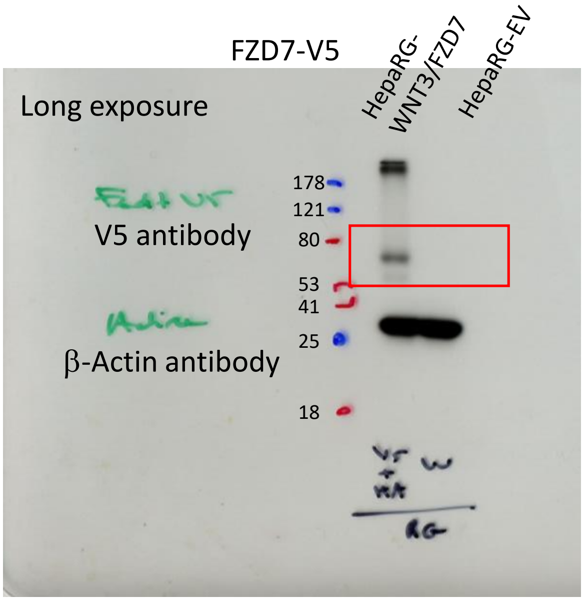

Figure 1A middle panel

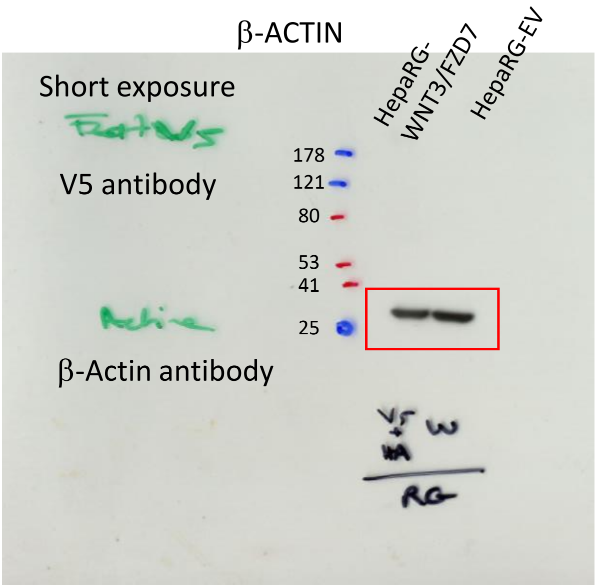

Figure 1A lower panel WNT3-HA

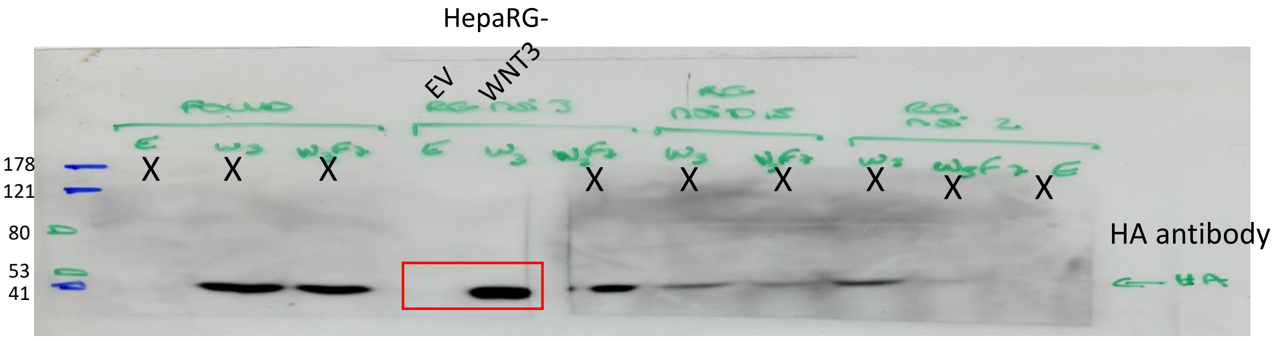

Supplement: S1 Raw Image — (PDF) [file pone.0332768.s003.pdf]
