# Supplementary material for: Frizzled 7 drives amplification of cancer stem-cell subpopulations and the aggressiveness and poor differentiation of human hepatocellular carcinoma
Source: PLoS One. 2025 Oct 7;20(10):e0332768. doi: 10.1371/journal.pone.0332768 (PMC12503320; doi:10.1371/journal.pone.0332768)
Supplement: S1 Table — 170 HCCs from the French and 329 from the TCGA cohorts were analyzed. Chi-squared test (p value). (+), overexpression. (DOCX) [file pone.0332768.s004.docx]

**Table S1. Prevalence of *FZD7* overexpression in the 499 HCCs depending of the differentiation of tumors.** 170 HCC from the French and 329 from the TCGA cohorts were analyzed. Chi-squared test (*p* value). ^(+)^, overexpression.

| Prevalence of stemness markers regarding clinico-biology, pathology | *FZD7*^(+)^ |
| --- | --- |
| Poor *vs.* moderate *vs.* good differentiation  (*p*) | 52% *vs.* 36% *vs.* 26%  ***(0.0001****)* |
