# Supplementary material for: Frizzled 7 drives amplification of cancer stem-cell subpopulations and the aggressiveness and poor differentiation of human hepatocellular carcinoma
Source: PLoS One. 2025 Oct 7;20(10):e0332768. doi: 10.1371/journal.pone.0332768 (PMC12503320; doi:10.1371/journal.pone.0332768)
Supplement: S2 Table — 140 HCCs were evaluable for survival rates in the French cohort. AFP, α-fetoprotein level; BCLC, Barcelona-Clinic Liver Cancer. (DOCX) [file pone.0332768.s005.docx]

**Table S2.** **Clinicopathological characteristics of human HCCs**. 140 HCC were evaluable for survival rates in the French cohort. AFP, α-fetoprotein level; BCLC, Barcelona-Clinic Liver Cancer.

| **Variables** | **Total (*n=*140)** |
| --- | --- |
| Age (years) Mean ± SD | 61.8 ± 10.6 |
| Sex Male | 83% |
| Etiology of the hepatopathy  HBV  HCV  Alcohol  NASH | 19.9%  32.2%  30.1%  17.8% |
| Cirrhosis | 53.7% |
| Child-Pugh score  A  B | 95%  5% |
| Tumor size (mm)  Mean (range)  HCCs > 50 mm | 56.2 (12 to 220)  39.6% |
| Number of HCC nodules  1  2  3  4  5 | 78.1%  12.4%  4.4%  2.2%  2.9% |
| BCLC-stage  0  A  B | 10%  72%  18% |
| Preoperative AFP level (ng/mL)  Mean  Median  Range  >200 ng/mL | 29,610  10  3 to 1,000,000  16% |
| Differentiation status  Well  Moderate  Poor | 40.9%  46%  13.1% |
| Microvascular invasion | 37.3% |
| Microscopic satellite nodules | 24.8% |
