# Supplementary material for: Frizzled 7 drives amplification of cancer stem-cell subpopulations and the aggressiveness and poor differentiation of human hepatocellular carcinoma
Source: PLoS One. 2025 Oct 7;20(10):e0332768. doi: 10.1371/journal.pone.0332768 (PMC12503320; doi:10.1371/journal.pone.0332768)
Supplement: S3 Table — Data are expressed as Spearman’s rank correlation coefficient (Rho) and p value. All these signatures correlated with each other and could not be identified as independent variables in multivariate analysis. (DOCX) [file pone.0332768.s006.docx]

**Table S3.** **Correlations between stemness signatures influencing overall survival (OS).** Data are expressed as Spearman’s rank correlation coefficient (Rho) and *p value*. All these signatures correlated with each other and could not be identified as independent variables in multivariate analysis.

|  | **Rho Spearman's coefficient of rank correlation** | ***p value*** |
| --- | --- | --- |
| *EPCAM*^(+)^ *vs.* *FZD7*^(+)^/*NANOG^(+)^* | 0.181 | *p<*0.003 |
| *EPCAM*^(+)^ *vs.* *FZD7*^(+)^/*CD133*^(+)^ | 0.429 | *p<*0.0001 |
| *EPCAM*^(+)^ *vs.* *EPCAM*^(+)^/*NANOG*^(+)^ | 0.531 | *p<*0.0001 |
| *EPCAM*^(+)^ *vs.* *NANOG*^(+)^/*CD133*^(+)^ | 0.324 | *p=*0.0001 |
| *FZD7*^(+)^/*NANOG^(+)^* *vs.* *FZD7*^(+)^/*CD133*^(+)^ | 0.409 | *p<*0.0001 |
| *FZD7*^(+)^/*NANOG^(+)^* *vs.* *EPCAM*^(+)^/*NANOG*^(+)^ | 0.513 | *p<*0.0001 |
| *FZD7*^(+)^/*NANOG^(+)^* *vs. NANOG*^(+)^/*CD133*^(+)^ | 0.661 | *p=*0.0001 |
| *FZD7*^(+)^/*CD133*^(+)^ *vs.* *EPCAM*^(+)^/*NANOG*^(+)^ | 0.399 | *p<*0.0001 |
| *FZD7*^(+)^/*CD133*^(+)^ *vs.* *NANOG*^(+)^/*CD133*^(+)^ | 0.682 | *p<*0.0001 |
| *EPCAM*^(+)^/*NANOG*^(+)^ *vs.* *NANOG*^(+)^/*CD133*^(+)^ | 0.638 | *p<*0.0001 |
