# Supplementary material for: Frizzled 7 drives amplification of cancer stem-cell subpopulations and the aggressiveness and poor differentiation of human hepatocellular carcinoma
Source: PLoS One. 2025 Oct 7;20(10):e0332768. doi: 10.1371/journal.pone.0332768 (PMC12503320; doi:10.1371/journal.pone.0332768)
Supplement: S4 Table — 415 HCCs (140 from the French and 275 from the TCGA cohorts) were analyzed. Cox proportional-hazards regression in univariate analysis; hazard ratios (HR) with 95% confidence intervals (CI). p value < 0.05 was considered as significant (in bold). Stemness signatures correlated between each other by Spearman's coefficient of rank correlation (rho, p value). (+), overexpression. (DOCX) [file pone.0332768.s007.docx]

**Table S4. Correlation between survival rates and the overexpression of FZD7, NANOG and CD133 stemness markers in HCCs.** 415 HCC (140 from the French and 275 from the TCGA cohorts) were analyzed. Cox proportional-hazards regression in univariate analysis; hazard ratios (HR) with 95% confidence intervals (CI). *p* value < 0.05 was considered as significant (in bold). Stemness signatures correlated between each other by Spearman's coefficient of rank correlation (rho, *p* value). ^(+)^, overexpression.

| **Univariate analysis of survival rates regarding variables *vs.* rest** | **Overall survival (OS)** | | **Recurrence-free survival (RFS)** | | **Post-recurrence survival** | | |
| --- | --- | --- | --- | --- | --- | --- | --- |
|  | HR (95% CI) | *p* | HR (95% CI) | *p* | HR (95% CI) | *p* |  |
| FZD7^(+)^ | 1.32 (0.92 – 1.90) | *0.13* | 1.12 (0.85 – 1.48) | *0.41* | 1.38 (0.92 – 2.07) | *0.12* |  |
| NANOG^(+)^ | 1.26 (0.86 – 1.85) | *0.24* | 1.12 (0.84 – 1.51) | *0.43* | 1.35 (0.88 - 2.05) | *0.16* |  |
| CD133^(+)^ | 1.35 (0.88 – 2.06) | *0.16* | 1.19 (0.85 – 1.66) | *0.32* | 1.46 (0.92 – 2.32) | *0.10* |  |
| FZD7^(+)^/NANOG^(+)^ | 1.40 (0.84 – 2.34) | *0.19* | 1.14 (0.76 – 1.72) | *0.51* | 1.58 (0.91 – 2.74) | *0.10* |  |
| FZD7^(+)^/CD133^(+)^ | 2.02 (1.27 – 3.21) | ***0.003*** | 1.22 (0.81 – 1.84) | *0.34* | 2.16 (1.29 – 3.62) | ***0.003*** |  |
| NANOG^(+)^/CD133^(+)^ | 1.40 (1.05 – 3.65) | ***0.03*** | 1.41 (0.82 – 2.42) | *0.21* | 1.86 (0.93 – 3.70) | *0.07* |  |
