# Supplementary material for: Frizzled 7 drives amplification of cancer stem-cell subpopulations and the aggressiveness and poor differentiation of human hepatocellular carcinoma
Source: PLoS One. 2025 Oct 7;20(10):e0332768. doi: 10.1371/journal.pone.0332768 (PMC12503320; doi:10.1371/journal.pone.0332768)
Supplement: S5 Table — Upper panel: The percentage of EpCAM or CD90 positive cells was assessed by FACS in Focus-EV, Focus-WNT3/FZD7 (12 independent experiments), Huh7-EV and Huh7-WNT3/FZD7 (3 independent experiments in duplicate). The percentage of CD133 positive cells was assessed by IF in Focus-EV and Focus-WNT3/FZD7. Ten random fields were analyzed in three independent experiments. Lower panel: The percentage of EpCAM(+) cells was also evaluated in Focus-EV and Focus-WNT3/FZD7, after shRNA-mediated invalidation of FZD7 (5 independent experiments). ND, non-determined. p value < 0.05 was considered as significant (in bold). shSCR, sh scramble. (DOCX) [file pone.0332768.s008.docx]

**Table S5. Expression of stemness-associated markers in WNT3/FZD7-overexpressing Focus and Huh7 cells**. Upper panel: The percentage of EpCAM or CD90 positive cells was assessed by FACS in Focus-EV, Focus-WNT3/FZD7 (12 independent experiments), Huh7-EV and Huh7-WNT3/FZD7 (3 independent experiments in duplicate). The percentage of CD133 positive cells was assessed by IF in Focus-EV and Focus-WNT3/FZD7. Ten random fields were analyzed in three independent experiments. Lower panel: The percentage of EpCAM^(+)^ cells was also evaluated in Focus-EV and Focus-WNT3/FZD7, after shRNA-mediated invalidation of FZD7 (5 independent experiments). ND, non-determined. *p* value < 0.05 was considered as significant (in bold). shSCR, sh scramble.

| **% of positive cells** | **EpCAM** | **CD90** | **CD133** |
| --- | --- | --- | --- |
| Focus-EV | 10.4 ± 3.1 | 8.3 ± 3.0 | 55.6 ± 6.7 |
| Focus-WNT3/FZD7 | 14.8 ± 4.4 | 10.7 ± 3.3 | 51.5 ± 7.7 |
| Paired *t-*test, *p value* | ***0.001*** | ***0.006*** | *0.19* |
| Huh7-EV | 24.3 ± 11.8 | 1.1 ± 0.9 | ND |
| Huh7-WNT3/FZD7 | 37.1 ± 16.2 | 1.4 ± 1.1 | ND |
| Paired *t-*test, *p value* | ***0.01*** | ***0.01*** | ND |
|  |  |  |  |
| **% of positive cells** | **EpCAM** | **CD90** | **CD133** |
| Focus-EV + shSCR | 8.4 ± 0.2 | ND | ND |
| Focus-EV + shFZD7 | 5.2 ± 0.9 | ND | ND |
| Paired *t-*test, *p value* | ***0.01*** | ND | ND |
| Focus-WNT3/FZD7 + shSCR | 13.8 ± 1.8 | ND | ND |
| Focus-WNT3/FZD7 + shFZD7 | 10.2 ± 1.8 | ND | ND |
| Paired *t-*test, *p value* | ***0.01*** | ND | ND |
